# Association of telomere length and telomerase methylation with n-3 fatty acids in preschool children with obesity

**DOI:** 10.1186/s12887-020-02487-x

**Published:** 2021-01-07

**Authors:** Xuanyi Liu, Xiaozhou Liu, Qiaoyu Shi, Xiuqin Fan, Kemin Qi

**Affiliations:** Laboratory of Nutrition and Development, Beijing Pediatric Research Institute, Beijing Children’s Hospital, Capital Medical University, National Center for Children’s Health, No.56 Nan-li-shi Road, Beijing, 100045 China

**Keywords:** Child obesity, Leukocyte telomere length, Telomerase reverse transcriptase, DNA methylation, N-3 fatty acids

## Abstract

**Background:**

Telomeres play a crucial role in cellular survival and its length is a predictor for onset of chronic non-communicable diseases. Studies on association between telomeres and obesity in children have brought discrepant results and the underlying mechanisms and influential factors are to be elucidated. This study aimed to investigate changes in telomere length and telomerase reverse transcriptase (TERT) DNA methylation, and further to determine their correlation with n-3 polyunsaturated fatty acids (PUFAs) in preschool children with obesity.

**Methods:**

Forty-six preschool children with obesity aged 3 to 4 years were included in the study, with equal numbers of age- and gender-matched children with normal weight as control. Leukocyte telomere length was determined by the ratio of telomeric product and single copy gene obtained using real-time qPCR. DNA methylation of TERT promoter was analyzed by bisulfite sequencing. Fatty acids in erythrocytes were measured by gas chromatography with a total of 15 fatty acids analyzed. The total saturated fatty acids (SFAs), total n-6 PUFAs, total n-3 PUFAs, and the ratio of arachidonic acid (AA) to docosahexaenoic acid (DHA) were calculated. Then the correlation between leukocyte telomere length, TERT promoter methylation and fatty acids was determined.

**Results:**

In preschool children with obesity, leukocyte telomeres were shortened and had a negative association with the body mass index. The methylated fractions in 13 of 25 CpG sites in the TERT promoter were increased by approximately 3 to 35% in the children with obesity compared to the normal weight children. Erythrocyte lauric acid and total SFAs, lenoleic acid and total n-6 PUFAs were higher, and DHA was lower in the children with obesity than those in the children with normal weight. Correlative analysis showed that leukocyte telomere length had a positive association with total SFAs and DHA, and a negative association with the AA/DHA ratio. However, no association between erythrocyte DHA and the TERT promoter methylation was found.

**Conclusion:**

These data indicate that the reduced body DHA content and increased AA/DHA ratio may be associated with shortened leukocyte telomeres in child obesity, which is probably not involved in the TERT promoter methylation.

**Supplementary Information:**

The online version contains supplementary material available at 10.1186/s12887-020-02487-x.

## Background

The rapidly increased prevalence of childhood obesity and its associated comorbidities including obstructive sleep apnea syndrome, psychological problems, cardiovascular diseases, type 2 diabetes, cancers, etc., are seriously threatening short-term and long-term health [1]. The underlying mechanisms for the obesity associated complications are attributable to the chronic inflammation, formation of reactive oxygen species (ROS), lipid peroxidation, and resulted DNA instability or damage [2, 3]. Telomeres, specialized DNA-protein structures located at the ends of eukaryotic chromosomes, have been found to be negatively correlated to obesity in length, being with an important heterogeneity [4, 5]. In children, studies have brought discrepant results, with some showing that obesity is related to shorter telomeres [6–8] but others finding no association [9, 10].

Telomeres are composed of repetitive DNA with highly conserved sequences (5′-TTAGGG-3′), and associated proteins [11], and play a crucial role in cellular survival, by maintaining chromosome stability and limiting progressive loss of genomic information caused by semi-conservative replication of DNA during the cycle of cell division [12, 13]. Telomere maintenance requires the active telomerase that is a ribonucleic acid-protein complex (RNP) composed of a single long non-coding telomerase RNA (TER), telomerase reverse transcriptase (TERT), and other proteins that vary among organisms, and functions to add telomeric repeat DNA to chromosome ends [14, 15]. It has been demonstrated that telomerase activity is mostly undetectable in somatic cells several weeks after birth, and high in cells with high generative potentials, such as germ line cells, hematopoietic and few types of stem cells, most cancer cells, and during embryonic development [16–18]. Mutations in many of the core telomerase RNP subunits, promoter elements, as well as the maturation factors lead to telomerase deficiency diseases such as dyskeratosis congenita or aplastic anemia, while its aberrant upregulation is a prerequisite for the immortal phenotype of most cancer cells [19–22]. In cancers, DNA hypermethylation with the TERT promoter is a prevalent telomerase-activating mechanism that can act independently of or in conjunction with promoter mutations [19, 20].

Cumulative evidences show that telomere length can be affected by many factors, such as age, gender, genetic variation, physiological stress, lifestyle factors (smoking, obesity, and lack of exercise), environmental exposure to carcinogens, and diseases (cancer, etc.) [14, 23]. Most of the telomere loss happens in the first 4 years of age, plateauing by age 4, with the greatest amount of attrition within the first years of life [18], followed by a slower attrition rate throughout adulthood. In obesity, telomere length is negatively associated with body weight and fat mass in American Indians, independently of chronological age, lifestyle factors and obesity-related inflammation or comorbidities [4]. Regarding the effects of diet and nutrients, most studies indicate that a healthy diet characterized by a high intake of dietary fiber and unsaturated lipids exerts a protective role on telomere health, whereas high consumption of sugar and saturated lipids accelerates telomere attrition [24]. National Health and Nutrition Examination Survey indicated that higher mineral and vitamin consumption is associated with longer telomeres among adults in the United States [25]. Vitamin D supplementation is found to be able to increase peripheral lymphocyte telomerase activity in overweight African Americans [26].

Since the 1960’s, indiscriminate recommendations have been made to substitute vegetable oils (high in n-6 PUFAs and low in n-3 PUFAs) for saturated fats, resulting in the increased ratio of n-6/n-3 PUFAs. This dietary fat change has been considered to be related to the increased prevalence of chronic non-communicable diseases including obesity, diabetes, cardiovascular diseases in modern society [27]. Under intake of n-3 PUFAs and its imbalanced ratio to n-6 PUFAs in early life may be an important determinant for adipocyte differentiation and growth and may relate to obesity pathogenesis in later life [28, 29]. Proposed mechanisms by which n-3 PUFAs improve body composition and counteract obesity-related metabolic changes include modulating lipid metabolism, adipokine expression and adipogenesis, alleviating adipose tissue inflammation and oxidative stress, and altering epigenetics [29–32]. Therefore, this study aimed to investigate changes in telomere length and TERT promoter DNA methylation, and further to determine their correlation with n-3 PUFAs in preschool children with obesity.

## Methods

### Subjects

A total of 50 preschool children with obesity aged 3 to 4 years and equal numbers of age- and gender-matched lean (normal weight) children were recruited in the study, during the yearly routine health examination in residential community in Beijing. After reviewing the documents and 4 children in each group excluded due to failure in blood sample drawn, 46 obese children and equal numbers of matched lean normal children were included in a final study. The detailed information was recorded on children’s height and body weight, feeding after birth, maternal age at birth, maternal education marital status, etc. Obesity was defined as having a body mass index (BMI) in the ≥ 2SD based on the WHO Multicentre Growth Reference Chart [33]. Children who had metabolic, endocrine and hereditary disorders, and diseases treated with glucocorticoids were excluded. Heparin-anticoagulant peripheral blood samples were collected. After centrifugal separation, the plasma was used for biochemical parameters of health examination, and the blood cells were stored at − 80 °C for determination of telomere length and TERT DNA methylation. The Institutional Review Board and Committee on Human Research at Beijing Children’s Hospital approved the study (No. 2016–3). The mothers or other legal guardians gave written consent for their participation and their children’s participation.

### Determination of telomere length

Determination of telomere length was ascertained using the real-time qPCR on a CFX96 Touch TM Real-Time PCR Detection System (Bio-Rad), based on extracted genomic DNA from the peripheral leukocytes. Telomere length was measured by the ratio of telomeric DNA product to single copy gene (T/S ratio), which was calculated by 2^-(∆C t t– ∆C t s)^/mean of all plates 2^-(∆C t t– ∆C t s)^ = 2^-∆∆C t^/mean 2^-∆∆C t^ [34]. The primers for the telomere PCR were tel. 1: 5′-GGTTTTTGAGGGTGAGGGTGAGGGTGAGGGTGAGGGT-3′, and tel. 2: 5′-TCCCGACTATCCCTATCCCTATCCCTATCCCTATCCCTA-3′, and the primers for the single copy gene human β-globin were Hbg1: 5′-GCTTCTGACACAACTGTGTTCACTAGC-3′, Hbg2: 5′-CACCAACTTCATCCACGTTCACC-3′. Each reaction was performed in the final volume of 20 μl. The thermocycle program consists of an initial hot start cycle at 95 °C for 30s, followed by 40 cycles at 95 °C for 5 s, 60 °C for 15 s and 72 °C for 10 s.

### Bisulfite conversion and sequencing

**Fig. 1:**
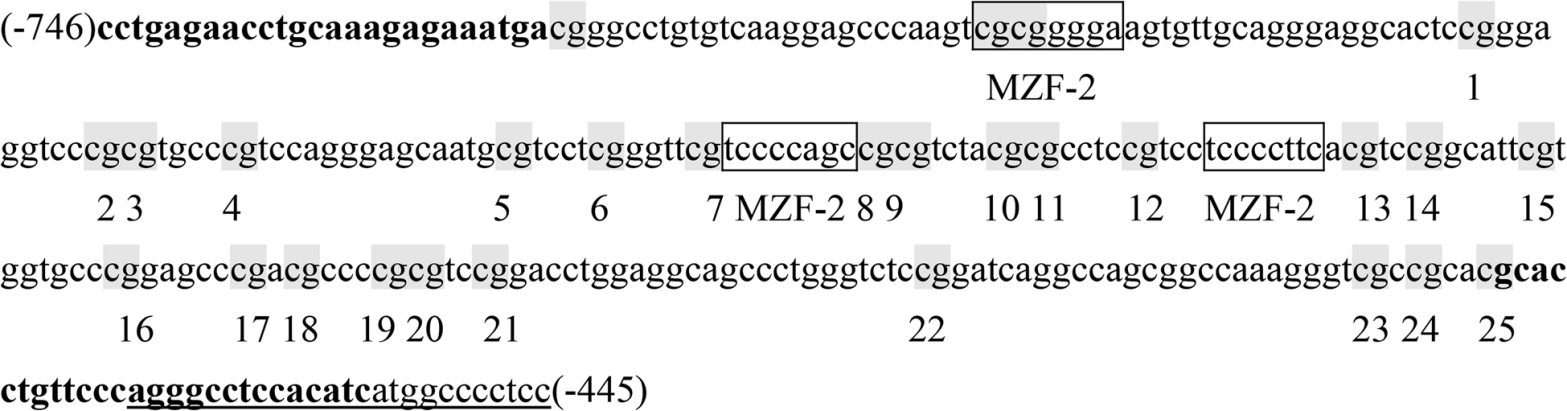
Primer sequences of the TERT promoter. The examined TERT promoter region includes nucleotides (nts) 1,254,147–1,253,148 and spans 25 CpGs within nts − 746 to − 445 (positions are given relative to the transcription start site), containing 3 binding sites (boxed) for myeloid-specific zinc finger protein 2 (MZF2). CpG sites are labeled with shadow and numbered from 1 to 25. Primers were shown in bold and the primer outer R was underlined

The examined TERT promoter region includes nucleotides (nts) 1,254,147–1,253,148 and spans 25 CpGs within nts − 746 to − 445 [positions are given relative to the transcription start site] (Fig. 1). The obtained sequence data have been submitted to the GenBank database (http://www.ncbi.nlm.nih.gov) under accession NC_000005, and contain binding sites for myeloid-specific zinc finger protein 2 (MZF2). DNA methylation of the promoter was analyzed by bisulfite sequencing. Genomic DNA was isolated and purified from the peripheral leukocytes in children with obesity and normal weight (*n*=46 each), using a TIANamp Micro DNA Kit (Tiangen Biotech, Beijing, China), and then was quantified and quality assessed by nanodrop ND-1000. Genomic DNA bisulfate conversion was conducted using the EZ DNA MethylationTM Kit (Cat. No. D5001, ZYMO Research, USA). The methyl-modified DNA was amplified by nested PCR and the products were sequenced directly. Specific primers for the TERT promoter were as follows: F, 5′- TTTGAGAATTTGTAAAGAGAAATGA-3′; inner R, 5′- AATATAAAAACCCTAAAAACAAATAC-3′; outer R, 5′- AAAAAAACCATAATATAAAAACCCT-3′. DNA methylation was calculated from the amplitude of cytosine and thymine within each CpG dinucleotide, C/(C+T), as described by Lewin et al. [35].

### Analysis of fatty acid profiles in erythrocytes

Fatty acids in erythrocytes were measured by gas chromatography (GC) in children with obesity and normal weight (*n*=20 each) included for examination of the TERT promoter methylation. Fatty acid methyl esters (FAMEs) from red blood cells were prepared according to a modified method of Lepage [36, 37]. Briefly, 200 μl of erythrocytes was added to 2 ml of a mixture solution of methanol n-hexane (4:1, vol/vol) containing C15:0 (an internal standard) and 2 μl of BHT (20 mM) to prevent lipid oxidation, and then 0.2 ml of acetyl chloride was slowly added. After heating at 100 °C for 1 h, 5 ml of a 6% K2CO3 solution was added to the tube, mixed on a vortex and centrifuged, and the clear n-hexane top layer containing FAMEs was transferred to a GC autosampler vial for analysis. FAMEs were analyzed according to our previously described method on an Agilent 6890 N GC system, and the quantity of fatty acids was expressed as the percent (%) (wt/wt) of the total fatty acids [38].

### Statistical analysis

Based on the estimated sample size (from 19 to 192) with α=0.05 and β=0.1, and changes in telomere length with obesity published, a total of 50 obese children and equal numbers of age- and gender-matched children with normal weight were recruited in the study. Analyses were performed using the GraphPad Prism 8.0. The Lilliefors test was used to evaluate whether the data is normally distributed, where *P*> 0.05 indicates normally distribution. Two-tailed t-test was used for the normally distributed data and two-sided Wilcoxon rank-sum test was used for the non-normally distributed data to analyze the differences between two groups. The Pearson correlation was used to determine the correlations between two parameters. All data were expressed as means ± SD, and *P*< 0.05 was considered to be statistically significant.

## Results

### Changes in telomere length in children with obesity

**Fig. 2:**
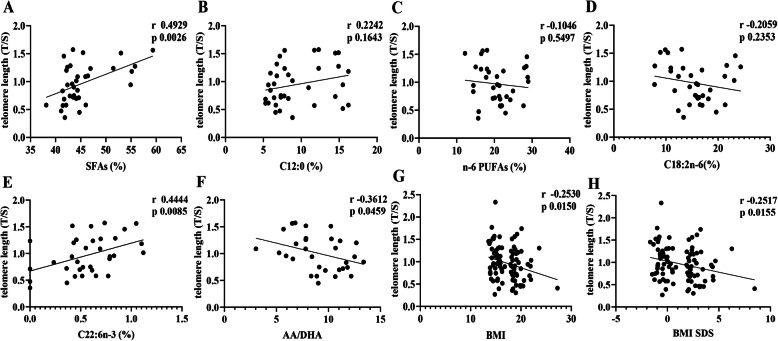
Associations of telomere length with erythrocyte fatty acid compositions and BMI. Leukocyte telomere length in children with obesity and normal weight (*n*=46 each) was determined using the real-time qPCR, and expressed as the ratio of telomeric product and single copy gene obtained. Meanwhile, fatty acids in erythrocytes (*n*=20 each group) were measured by gas chromatography, and their quantity was expressed as the percent (%) of the total fatty acids. The associations of telomere length with fatty acids, BMI and BMI SDS were analyzed by the Pearson correlation

The characteristics of subjects in this study were presented in Table 1. As expected, the leukocyte telomere length was reduced in children with obesity compared to children with normal weight (*P*< 0.05). Correlative analysis showed a negative association between telomere length and the BMI as well as BMI SDS (*P*< 0.05) (Fig. 2). Additionally, children with obesity had higher birth weight and birth BMI and lower breastfeeding rates within the first 3 months postnatally (*P*< 0.05). No correlation between age as months and telomere length was indicated (Additional file 1).

**Table 1:** Clinical characteristics and leukocyte telomere length in children with obesity versus normal weight

	Obese (*n*=46) (F=20, M=26)	Control (*n*=46) (F=21 M=25)	P
Age (years)	3.31±0.47	3.25±0.66	0.5884
BMI (kg/m^2^)	19.52±1.71^a^	15.05±0.80	< 0.0001
BMI SDS	2.93±1.23^a^	-0.41±0.59	< 0.0001
Weight at birth (g)	3527.19±422.21^a^	3287.81±448.19	0.0084
Length at birth (cm)	50.33±1.54	49.87±1.51	0.1497
BMI at birth (kg/m^2^)	13.92±1.26^a^	13.46±2.16	0.0069
Gestational age at delivery (wk)	38.98±1.47	38.75±1.48	0.4514
Breast feeding within 3 months	19/40^a^	31/42	0.0162
Breast feeding within 6 months	13/37	22/40	0.0803
Leukocyte telomere length (T/S)	0.88±0.36^a^	1.05±0.39	0.0433

^a^Significant differences between the obese and control children

### Changes in DNA methylation of the TERT promoter in children with obesity

As shown in Table 2, a total of 25 CpG sites at the TERT promotor were determined for DNA methylation. The methylated fractions in 13 CpG sites were significantly increased by approximately 3 to 35% in children with obesity compared with children with normal weight (*P*< 0.05), including 7 CpG sites located at areas for binding MZF2. Correspondently, the averaged methylation fraction was increased in children with obesity compared to children with normal weight (*P*< 0.05). No association of CpG methylation fractions with telomere length was found (Additional file 1).

**Table 2:** Changes in methylation fraction of CpG sites of the TERT promoter in children with obesity

CpG sites	Obese (*n*=46)	Control (*n*=46)	t	P
1	0.71±0.05	0.70±0.03	0.773	0.4414
2	0.72±0.05	0.71±0.03	1.864	0.0653
3	0.46±0.08	0.45±0.03	1.152	0.2523
4	0.72±0.06^a^	0.70±0.03	2.434	0.0168
5	0.60±0.07	0.59±0.04	0.696	0.4878
6	0.33±0.06	0.32±0.05	1.034	0.3038
7	0.50±0.05	0.49±0.04	1.747	0.0838
8	0.55±0.06	0.54±0.04	1.757	0.0822
9	0.54±0.08^a^	0.51±0.04	2.207	0.0454
10	0.63±0.06^a^	0.60±0.03	2.745	0.0072
11	0.58±0.08^a^	0.55±0.04	2.434	0.0168
12	0.66±0.05^a^	0.64±0.04	2.023	0.0458
13	0.27±0.08	0.26±0.04	1.010	0.3151
14	0.56±0.08^a^	0.52±0.04	3.030	0.0310
15	0.38±0.07^a^	0.35±0.04	2.375	0.0196
16	0.41±0.09^a^	0.38±0.04	2.241	0.0270
17	0.31±0.09^a^	0.27±0.04	2.701	0.0082
18	0.34±0.10	0.31±0.04	1.956	0.0533
19	0.47±0.10^a^	0.43±0.05	2.869	0.0051
20	0.39±0.12^a^	0.33±0.04	2.946	0.0041
21	0.40±0.09	0.37±0.04	1.955	0.0535
22	0.21±0.09^a^	0.18±0.03	2.894	0.0048
23	0.23±0.14^a^	0.17±0.06	2.929	0.0044
24	0.36±0.12	0.33±0.06	1.071	0.2872
25	0.19±0.11	0.16±0.06	1.473	0.1447
Average	0.47±0.06^a^	0.44±0.03	0.405	0.0010

^a^Significant differences between the obese and control children

### Differences in erythrocyte fatty acids between children with obesity and normal weight

The erythrocyte fatty acid compositions in two groups of children were presented in Table 3. Lauric acid (C12:0) and total saturated fatty acids, lenoleic acid (C18:2n-6) and total n-6 PUFAs were higher in children with obesity than those in children with normal weight (*P*< 0.05). Whereas, docosahexaenoic acid (DHA, C22:6n-3) was decreased in children with obesity, resulting in an increased ratio of arachidonic acid (AA, C20:4n-6) to DHA, compared to children with normal weight (*P*< 0.05).

**Table 3:** Changes in erythrocyte fatty acids in children with obesity

Variables	Obese (*n*=20)	Control (*n*=20)	t	P
**Total SFAs**	46.99±5.70^a^	42.62±2.40	3.1570	0.0031
C8:0	3.81±2.61	3.23±1.76	0.8174	0.4188
C10:0	0.18±0.16	0.37±0.32	2.2308	0.0265
C12:0	9.33±3.60^a^	4.92±0.92	5.3060	< 0.0001
C14:0	0.04±0.09	0.01±0.02	1.6880	0.0994
C16:0	23.26±1.62	24.00±2.22	1.1960	0.2393
C18:0	10.36±1.67	10.09±1.19	0.5833	0.5631
**Total MUFAs**	25.68±3.21	25.36±2.78	0.5562	0.5808
C16:1	0.95±0.50	1.09±0.29	0.3322	0.7416
C18:1	24.74±3.21	24.27±2.70	0.4996	0.6203
**Total n-6 PUFAs**	19.40±3.85^a^	22.46±5.29	2.0930	0.0432
C18:2n-6	14.09±3.91^a^	17.11±4.55	2.2490	0.0304
C20:4n-6	5.28±1.61	5.31±1.23	0.0736	0.9417
C22:5n-6	0.04±0.07	0.05±0.09	0.4042	0.6883
**Total n-3 PUFAs**	7.92±2.64	9.44±4.61	1.3700	0.1787
C18:3n-3	0.03±0.07	0.00±0.00	2.1580	0.0373
C20:5n-3	3.77±1.59	3.59±1.74	0.3386	0.7368
C20:4n-3	3.66±1.87	5.16±3.13	1.8380	0.0739
C22:6n-3	0.46±0.33^a^	0.80±0.46	2.6650	0.0112
**AA/DHA**	10.35±1.97^a^	7.68±2.46	3.4400	0.0016
**Total n-6/n-3 PUFAs**	2.69±0.93	3.17±2.08	0.9510	0.3476

^a^Significant differences between the obese and control children

### Correlative analysis between telomere length, TERT promoter methylation and erythrocyte fatty acids

As shown in Fig. 2, the leukocyte telomere length was positively associated with concentrations of total SFAs and DHA in erythrocytes (*P*< 0.05), and negatively with the AA/DHA ratio (*P*< 0.05). However, no correlations between the TERT promoter methylation and erythrocyte fatty acid concentrations were indicated (Additional file 1).

## Discussion

A systematic review and meta-analysis has demonstrated a negative correlation between adult obesity and telomere length with weak to moderate statistical significance for the main research, and an important heterogeneity [5]. In child population, studies investigating telomere length and obesity, and their causal relationship proved inconclusive [6–10]. In this study, in Chinese preschool children with obesity, leukocyte telomeres were shown being shortened and had a negative association with the BMI. This may increase the risk of metabolic complications in later life, because leukocyte telomere length has been found to predict onset of cardiometabolic diseases in adults, specifically diabetes mellitus and cardiovascular disease [39–41]. Also, the shorter telomere length in preschool age is associated with obesity at age 9 in Latino Children [9].

Although the molecular mechanisms by which obesity affects telomere length have not been described clearly, current research findings suggest that the shortened telomere was probably caused by the increased chronic inflammation and oxidative stress in obesity [42]. It is reported that expression of TERT, the key enzyme for maintaining telomere length, induced by cytokines is organized through the PI3K/AKT and NF/kB signaling pathways [22]. In the present study, we found that DNA methylation fractions of the TERT promoter were increased in children with obesity. Epigenetic changes including DNA methylation have been recognized to be the key players in governing gene expression, and higher promoter DNA methylation may down-regulate gene expression [43, 44]. The data presented here suggested that DNA hypermethylation at the TERT promoter might reduce mRNA expression of TERT and its activity, and thus lead to telomere attrition in obesity. Consistently, hypermethylation of the TERT promoter alleles signals transcriptional repression of those alleles, leading to attenuation of TERT activation in cancer cells [45]. In contrast, many studies reported that DNA hypermethylation of the TERT promoter is positively related to telomerase activity in cancers [20, 46, 47].

The available evidence suggests that some antioxidant nutrients, the consumption of fruits and vegetables, and Mediterranean diet are mainly associated with longer telomeres [48]. With regard to macronutrients and telomeres, total protein and carbohydrates have not been clearly associated with telomere length; whereas the quality of carbohydrates, and particularly dietary fiber, may have a potential beneficial effect on telomere health [49, 50]. The effects of dietary fats on telomeres have been studied in more detail than the other macronutrients. Although the overall relation of monounsaturated fatty acids and PUFAs with telomere length is inconsistent [48], most studies have demonstrated a positive relation between n-3 PUFAs and telomere length in adult chronic diseases [51–53]. Differently, a study on Chinese adults reported that n-6 PUFAs are positively, but n-3 PUFAs are negatively associated with leukocyte telomere [54]. Herein we found that erythrocyte DHA content was decreased in obese children, and had a positive association to leukocyte telomere length. Unfortunately, no association between erythrocyte DHA and DNA methylation of the TERT promoter was shown, although DHA functions to regulate DNA and histone methylation through affecting methyl group metabolism [31, 32, 55]. Therefore, the mechanisms by which DHA affects telomere length may reside in its anti-inflammatory or anti-oxidative stress effects [29, 30], but is independent of its function in epigenetic modification.

With respect to saturated fat, it has been reported that women with SFAs intake in the lowest compared to highest quartile had significantly longer telomeres, and that in men, high total fat, SFAs, and butter intake was inversely associated with telomere length [56, 57]. In contrast, we found that erythrocyte total SFAs were increased in children with obesity, and were positively correlated with telomere length. The underlying reasons could not be answered herein. It may be involved in more total fat and SFAs required for infants and toddlers than older children and adults. High fat diets are important during early life because of very high energy needed for growth and for the rapid development of the nervous system, and fat restrictions may affect body composition and increase obesity risk in later life [58]. To note, in addition to fatty acids, many other factors reported can affect telomere length, such as age, gender, physiological and psychological stress, sedentary lifestyle, exposure to environmental carcinogens, etc. [14, 23]. Thus, more researches are needed to clarify the underlying mechanisms for shortened telomere length in the future.

The duration of obesity could have a significant influence on the observed changes in telomeres. Unfortunately, the data after birth at fixed time points, reflecting the development of obesity, were not available and thus its effects on telomere length could not be investigated in the current study. This question should be highlighted as a future research perspective. Meanwhile, the severity of obesity may also affect telomeres. It could be intriguing to divide the children into moderate obese and severe obese, but the smaller number could not allow further analysis and satisfactions. Future studies with a large sample size may be helpful for this hypothesis test.

## Conclusion

In summary, preschool children with obesity had shorter leukocyte telomeres, accompanied by DNA hypermethylation at the TERT promoter. A possible relationship between the shortened leukocyte telomeres and reduced body DHA content and increased AA/DHA ratio in obesity is suggested. However, the association of DHA with telomere length might not be involved in its modification on the TERT promoter DNA methylation.

## Supplementary Information

**Additional file 1.**

## Data Availability

The datasets used and/or analysed during the current study are available from the corresponding author on reasonable request.

## Abbreviations

AA: Arachidonic acid
BMI: Body mass index
DHA: Docosahexaenoic acid
FAMEs: Fatty acid methyl esters
MZF2: Myeloid-specific zinc finger protein 2
PUFAs: Polyunsaturated fatty acids
SFAs: Saturated fatty acids
TERT: Telomerase reverse transcriptase
