# Supplementary figures and images for: Association of telomere length and telomerase methylation with n-3 fatty acids in preschool children with obesity
Source: BMC Pediatr. 2021 Jan 7;21:24. doi: 10.1186/s12887-020-02487-x (PMC7788823; doi:10.1186/s12887-020-02487-x)

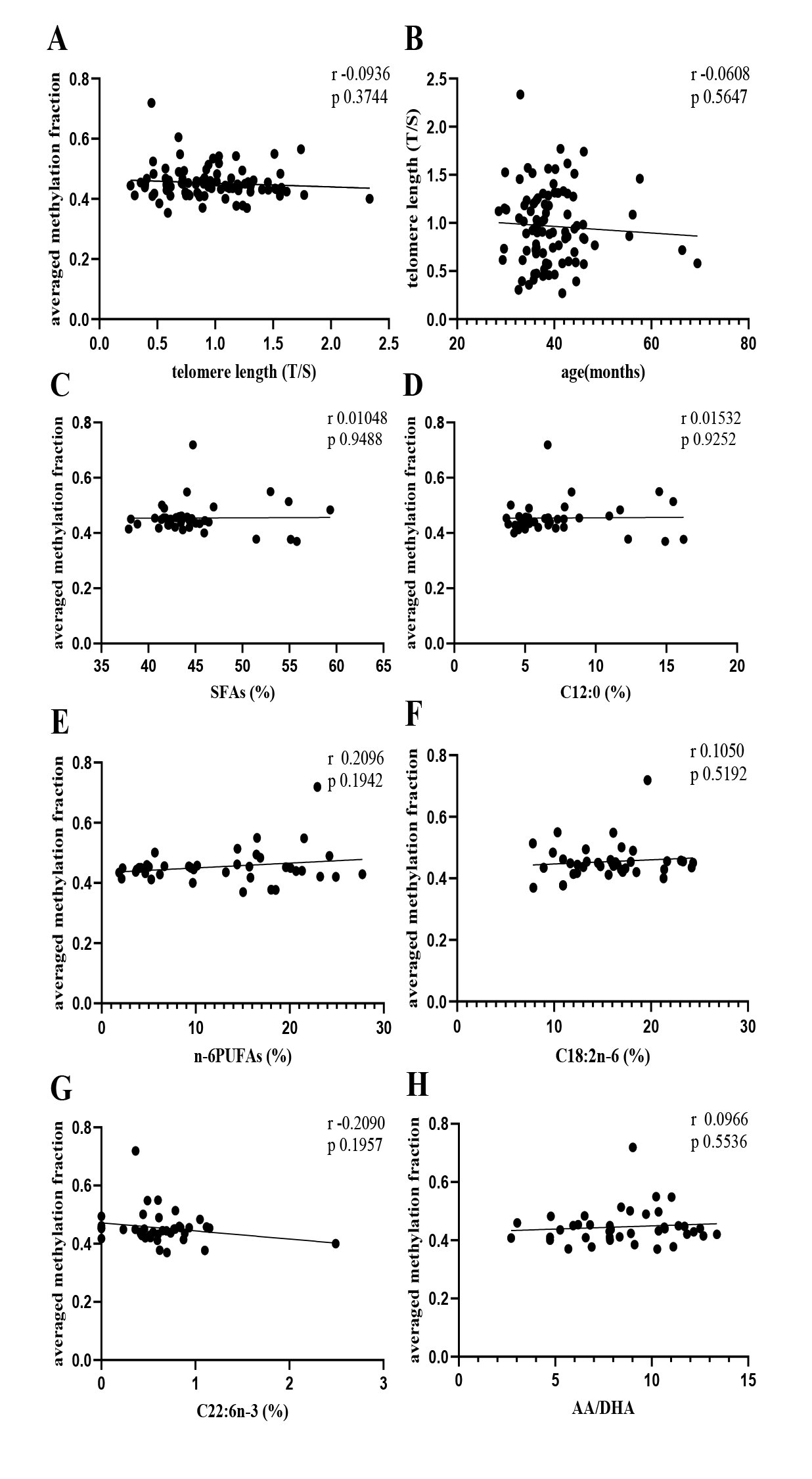

Supplement: Supplementary file 1 — Additional file 1. [file 12887_2020_2487_MOESM1_ESM.tif]
